# Sequential dual-site High-Definition transcranial Direct Current Stimulation (HD-tDCS) treatment in chronic subjective tinnitus: study protocol of a double-blind, randomized, placebo-controlled trial

**DOI:** 10.1186/s13063-019-3594-y

**Published:** 2019-08-01

**Authors:** E. Cardon, V. Van Rompaey, L. Jacquemin, G. Mertens, H. Vermeersch, I. Joossen, J. Beyers, O. M. Vanderveken, P. Van de Heyning, V. Topsakal, A. Gilles

**Affiliations:** 1 Department of Translational Neurosciences, Faculty of Medicine and Health Sciences, Campus Drie Eiken, Antwerp University, Antwerp, Belgium; 2 Department of Otorhinolaryngology – Head and Neck Surgery, Antwerp University Hospital, Wilrijkstraat 10, 2650 Edegem, Belgium; 3 Department of Education, Health & Social Work, University College Ghent, Ghent, Belgium

**Keywords:** Chronic tinnitus, Transcranial direct current stimulation, Neuromodulation, Randomized controlled trial

## Abstract

**Background:**

Chronic tinnitus is a highly prevalent symptom, with many patients reporting considerable effects of tinnitus on quality of life. No clear evidence-based treatment options are currently available. While counseling-based methods are valuable in some cases, they are not sufficiently effective for all tinnitus patients. Neuromodulation techniques such as high-definition transcranial direct current stimulation (HD-tDCS) are proposed to have positive effects on tinnitus severity but, to date, these effects have not been proven conclusively. The proposed trial will investigate the hypothesis that chronic tinnitus patients receiving HD-tDCS will report a positive effect on the impact of tinnitus on daily life, as compared to patients receiving sham stimulation.

**Methods:**

This study proposes a randomized, double-blind, placebo-controlled trial with parallel group design. A total of 100 chronic tinnitus patients will be randomly allocated to an experimental group or a sham group, with allocation stratified according to gender and tinnitus severity. Patient and researcher will be blinded to the patient’s allocation. Patients will undergo six sessions of sequential dual-site HD-tDCS of the left temporal area and the right dorsolateral prefrontal cortex. Evaluations will take place at baseline, immediately following treatment, and at three and six months after the start of the therapy. The primary outcome measure is the change in Tinnitus Functional Index (TFI) score. Secondary outcome measures include audiological measurements, cortical auditory evoked potentials, the Repeatable Battery for the Assessment of Neuropsychological Status adjusted for hearing-impaired individuals (RBANS-H), and supplementary questionnaires probing tinnitus severity and additional symptoms. By use of a linear regression model, the effects of HD-tDCS compared to sham stimulation will be assessed.

**Discussion:**

The objective of this study is to evaluate whether HD-tDCS can reduce the impact of tinnitus on daily life in chronic tinnitus patients. To date, published trials on the effects of HD-tDCS on tinnitus suffer from a lack of standardization and few randomized controlled trials exist. The proposed study will be the first adequately powered trial to investigate the effects of sequential dual-site HD-tDCS on tinnitus severity.

**Trial registration:**

ClinicalTrials.gov, NCT03754127. Registered on 22 November 2018.

**Electronic supplementary material:**

The online version of this article (10.1186/s13063-019-3594-y) contains supplementary material, which is available to authorized users.

## Background

Subjective tinnitus is commonly defined as the perception of sound in the absence of a corresponding external sound source. Approximately 10–20% of the population experience tinnitus [1, 2]. A segment of this patient group, amounting to 2–3% of the total adult population, describes their tinnitus as bothersome and interfering with everyday life [3]. Subjective tinnitus is often accompanied by non-specific symptoms such as annoyance, irritability, anxiety, depression, hearing problems, hyperacusis, insomnia, and concentration difficulties [4–6]. For some patients, tinnitus presents a severe burden that considerably affects quality of life. Due, in part, to considerable heterogeneity of the tinnitus patient population, no efficient evidence-based treatment option is currently available and personalized tinnitus therapy is essential [7]. In a specific subgroup of patients for whom tinnitus is accompanied by hearing impairment or single-sided deafness, hearing aids or cochlear implants are beneficial [8, 9]. Although counseling-based methods are proven to be effective for a large subgroup of patients, they sometimes only have modest effects on tinnitus severity and do not affect the underlying pathology [10, 11].

Tinnitus often develops as a result of damage to the cochlea [12]. Possible causes of this cochlear damage include sudden hearing loss, noise trauma, presbyacusis, and infections. However, hearing loss is not a prerequisite for the development of tinnitus and tinnitus perception seems to be regulated mainly by the central nervous system. Cochlear damage may lead to deafferentiation of auditory pathways and maladaptive plastic changes in wide brain networks comprising both cortical and subcortical areas [13]. Thus, tinnitus can be defined as an emergent property of multiple networks activated in parallel [14], with increased spontaneous activity and synchrony of affected neurons leading to its perception [15]. These alterations in brain activity might also be reflected in the cognitive deficits often observed in tinnitus patients [16]. Explorative research into these aspects of tinnitus, using auditory evoked potentials and cognitive tests that are adequately altered for hearing impaired individuals, might lead to a better characterization of different tinnitus subtypes [4, 17, 18].

Neuronal network activity can be modified by neuromodulation techniques such as transcranial direct current stimulation (tDCS). tDCS is a non-invasive and safe experimental therapy whereby the brain is stimulated with externally applied direct current electric fields in order to modify corticospinal excitability [19]. Effects of tDCS transcend the level of individual neurons, as the technique can influence brain-wide functional connectivity, network synchronization, and oscillatory activity [20]. Recently, the development of high-definition tDCS (HD-tDCS) has enabled researchers to stimulate brain regions with increased focality by using a higher number of smaller electrodes [21, 22]. Clinical applications of tDCS and HD-tDCS include pain relief, especially in fibromyalgia, the treatment of major depressive disorder, and tinnitus [23].

In tinnitus treatment, tDCS and HD-tDCS are mainly applied to two target regions: the left temporal area (LTA) and the right dorsolateral prefrontal cortex (rDLPFC) [24]. The LTA comprises the left auditory cortex, which shows hyperactivation in tinnitus patients [25]. The rDLPFC is involved in the integration of sensory and emotional aspects of tinnitus and is an essential part of the tinnitus distress network [26]. Overall, beneficial effects of tDCS of the rDLPFC have been reported and there are some indications of the benefit of tDCS of the LTA, but randomized controlled trials (RCT) on the effects of tDCS on tinnitus are severely lacking (for review, see [27]). In two recent studies reporting the efficacy of (HD-)tDCS of LTA and rDLPFC in tinnitus treatment, no difference in efficacy was found between the two target regions [24, 28]. Current evidence is too weak to make conclusive recommendations on the use of (HD-)tDCS in tinnitus treatment [23]. As such, there is a need for adequately powered double-blind randomized sham-controlled trials.

This study proposes a RCT with parallel group design, in which participants are randomly allocated to the experimental group or the sham group in a 1:1 ratio. This trial intends to investigate the superiority of HD-tDCS treatment over sham stimulation. It is hypothesized that tinnitus patients receiving six consecutive sessions of sequential dual-site HD-tDCS will report a positive effect on the impact of tinnitus on daily life, as compared to tinnitus patients receiving sham stimulation.

## Methods

### Study setting

Patients will be recruited at the tertiary tinnitus clinic of the Antwerp University Hospital (UZA). When tinnitus patients present themselves at the ear-nose-throat department, a thorough diagnostic evaluation takes place. A systematic medical history is obtained and coexistent symptoms (e.g. hearing loss, sleeping disorders, cervical tension or pain, or temporomandibular joint dysfunction) are enquired. Apart from the medical history, psychoacoustic characteristics of the tinnitus sound are quantified and the impact of tinnitus on quality of life is estimated via questionnaires. A thorough clinical exam containing micro-otoscopy, pure-tone audiometry, and psychoacoustic measures is performed. Together, these examinations will allow the evaluator to determine whether the patient meets the inclusion criteria for the study (Table 1). In case inclusion criteria are met, patients will be informed about the clinical trial and asked to participate.

**Table 1:** Inclusion and exclusion criteria

Inclusion	Exclusion
- Chronic (> 6 months) subjective tinnitus	- Somatic tinnitus
- 24 < TFI score < 90	- Pregnancy
- Hospital Anxiety and Depression Scale:	- Active middle ear pathology
• Depression subscale < 12	- Hearing implants
• Anxiety subscale < 12	- Known tumors in the head/neck region
- HQ score < 40	- Patient having already had any other tinnitus treatment within the last two months

*TFI* Tinnitus Functional Index, *HADS* Hospital Anxiety and Depression Scale, *HQ* Hyperacusis Questionnaire

### Eligibility criteria

Adult patients (male/female) with chronic (> 6 months), non-pulsatile subjective tinnitus will be included in the study (Table 1). Confounding effects of anxiety and depression will be controlled by using the Hospital Anxiety and Depression Scale (HADS). In case of uncertainty concerning the mental state or possible active psychiatric disorders, the patient will first be evaluated by the psychiatric diagnosis team of the Antwerp University Hospital.

### Interventions

**Fig. 1:**
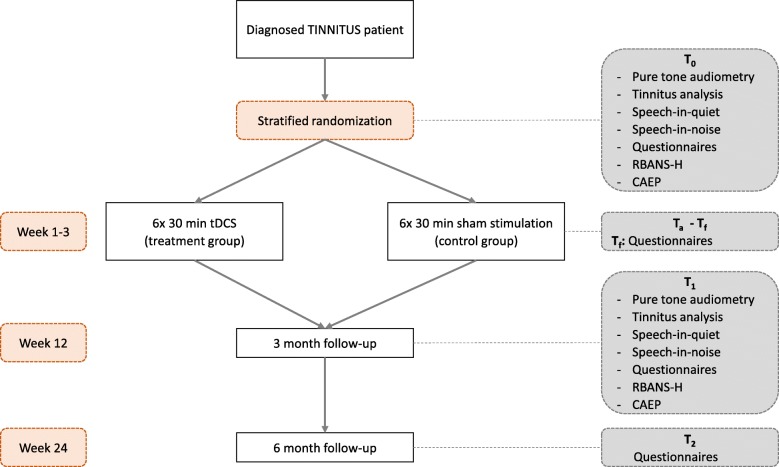
Visit and follow-up schedule. RBANS-H Repeatable Battery for the Assessment of Neuropsychological Status adjusted for hearing-impaired individuals, CAEP cortical auditory evoked potentials

**Fig. 2:**
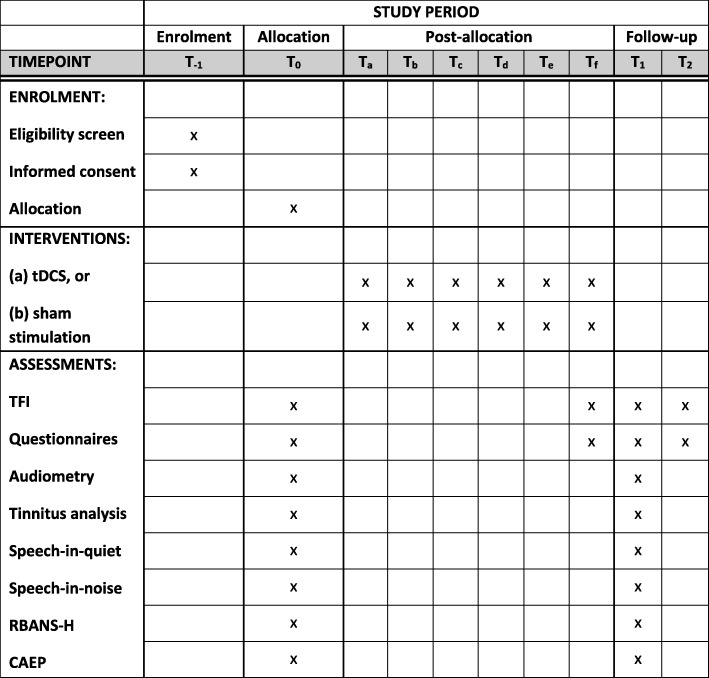
Schedule of enrolment, interventions, and assessments in accordance with the SPIRIT 2013 guidelines. tDCS transcranial direct current stimulation, TFI Tinnitus Functional Index, RBANS-H Repeatable Battery for the Assessment of Neuropsychological status adjusted for hearing-impaired individuals, CAEP cortical auditory evoked potentials

Participants are invited for a first clinical testing at the site (baseline measurement, T_0_). This testing consists of pure tone audiometry, psychoacoustic tinnitus analysis (loudness and pitch matching of the tinnitus), speech-in-quiet (SPIQ) and speech-in-noise (SPIN) measurements, questionnaires, and explorative research tests (Repeatable Battery for the Assessment of Neuropsychological Status, adjusted for hearing-impaired individuals [RBANS-H] and cortical auditory evoked potentials [CAEP]). The entire test protocol will take approximately 2.5–3 h. The schedule for patient visits and follow-ups is illustrated in Fig. 1. After the baseline measurements, patients are randomized into the HD-tDCS group or the sham control group. Figure 2 represents an overview of all interventions and outcome measures in accordance with the Standard Protocol Items: Recommendations for Interventional Trials (SPIRIT) 2013 guidelines [29] (Additional file 1).

When patients are enrolled in the study, six sessions of sequential dual-site HD-tDCS (or sham stimulation for the control group) are planned within three weeks’ time (twice per week, with at least one day between sessions: T_a_ - T_f_). Stimulation (or sham) is provided for 30 min each session. The electrodes are positioned at the rDLPFC and the LTA. The positioning is according to the 10/20 international system for EEG electrode placement. For the rDLPFC stimulation, the central anode will be placed at F4 and the adjoining cathodes at F2, F6, FC4, and AF4 [24]. For the LTA stimulation, the central anode is positioned at CP5 with adjoining cathodes at C5, TP7, CP3, and P5. A constant current of 2 mA is applied for 15 min at each site with a fade-in and fade-out of 20s, resulting in a total stimulation duration of 30 min. For the sham stimulation, constant current will only be applied for 20s as previously described [30]. The direct current is transmitted by means of five sintered silver/silver chloride (Ag/AgCl) ring electrodes with inner radius of 6 mm and outer radius of 12 mm and delivered by a battery-driven Soterix Medical 1 × 1 tDCS low-intensity stimulator and 4 × 1 multichannel stimulation adaptor (Soterix Medical Inc., New York, NY, USA) with a maximum output of 2 mA. The ring electrodes are stabilized using HD-Electrode holders hooked in the Soterix Medical HD-cap and filled with EEG electrode gel (Neurax, Bonheiden, Belgium) following the guidelines for 4 × 1 HD tDCS stimulation [22].

Immediately after the last HD-tDCS session (T_f_), the patient is instructed to fill in all questionnaires. A follow-up (T_1_) at three months after the baseline measurements will contain the full clinical study protocol: pure tone audiometry, tinnitus analysis, SPIQ/SPIN, questionnaires, RBANS-H, and CAEP. A second follow-up (T_2_) is planned at six months after baseline measurements, comprising only the complete set of questionnaires.

Except for mild itching or tingling sensations, headaches, or fatigue, no adverse effects of HD-tDCS treatment are expected. In case patients do report serious side effects, HD-tDCS treatment will be discontinued.

No concomitant tinnitus treatment will be allowed between baseline measurement and the three-month follow up. After the three-month time point, participants will be allowed to undergo additional treatment. If participants were allocated to the sham group, they will have the right to undergo an actual HD-tDCS treatment free of cost after the three-month time point.

### Outcomes

#### Primary outcome measure

The primary outcome is the change in the Tinnitus Functional Index (TFI) questionnaire from baseline to follow-up at three months. The TFI is a self-reported questionnaire assessing the impact of tinnitus on quality of life [31, 32]. The patient must answer 25 questions on a Likert scale in the range of 0–10. For questions 1 and 3, which are expressed in percentages, the Likert scale is transformed to a 0–10 scale post hoc. The total score is then calculated as the mean of all questions multiplied by 10 and expressed as a number between 0 and 100. Results of the TFI include the total score and the score on eight subscales: intrusiveness; sense of control; cognition; sleep disturbance; auditory difficulties; relaxation; quality of life; and emotional distress. A decrease of 13 points on the total TFI score is considered as a clinically relevant difference [32]. Based on previous studies, we expect mean TFI scores at baseline of approximately 50 [28].

#### Secondary outcome measures

- Tinnitus Questionnaire (TQ)

The Dutch validated version of the TQ is used to differentiate between emotional and cognitive distress, auditory difficulties, and self-experienced intrusiveness caused by tinnitus [33]. The total score can be in the range of 0–84, assigning a subject to a distress category: slight (score = 0–30, grade 1); moderate (score = 31–46, grade 2); severe (score = 47–59, grade 3); and very severe (score = 60–84, grade 4) [34].

- Speech, Spatial and Qualities of Hearing Scale-12 questionnaire (SSQ12)

The SSQ12 is a short form of the Speech, Spatial and Qualities of Hearing scale [35, 36]. The questionnaire is used in clinical research to measure several aspects of hearing ability, such as: speech comprehension in quiet and noise; localization of sound, distance, and movement; segregation; and listening effort. Responders rate their ability to do or experience the situation described in each question by marking a 1–10 scale (1 = not at all, 10 = perfectly). Scores of all 12 questions are averaged to obtain a global SSQ-12 score.

- Hospital Anxiety and Depression Scale (HADS)

The HADS is used to detect states of anxiety or depression [37]. Patients must answer a total of 14 questions, of which seven belong to the subscale “depression” and seven pertain to the subscale “anxiety.” Scores of 7 or less on each subscale indicate non-cases. Scores of 8–10 are borderline abnormal (borderline case), while scores of 11–21 are abnormal (case).

- Visual Analogue Scale (VAS)

The VAS enquires the mean loudness and maximum loudness of the tinnitus. The patient must score their tinnitus loudness on a scale of 0 (absence of tinnitus) to 100 (cannot be any louder).

- Hyperacusis Questionnaire (HQ)

The patient’s hypersensitivity to sound is investigated using the 14-item HQ [38]. The 14 questions assess three dimensions (attentional, social, and emotional). The answer categories are: “no” (score of 0 points); “yes, a little” (1 point); “yes, quite a lot” (2 points); and “yes, a lot” (3 points). A total score of 28 or more indicates clinically significant hyperacusis.

- Health Utilities Index (HUI23)

The HUI23 is a family of generic health profiles and preference-based systems for the purposes of measuring health status, reporting health-related quality of life (QoL), and producing utility scores [39]. The HUI23 comprises a 15-item questionnaire. The resulting total health-related QoL score ranges from 0.00 (dead) to 1.00 (perfect health).

- Pure-tone audiometry

Pure-tone linear audiometry will be performed according to current clinical standards (International Organization for Standardization [ISO] 8253–1:2010), using a two-channel AC-40 audiometer (Interacoustics, Assens, Denmark) in a soundproof booth. Air conduction thresholds will be measured at 125 Hz, 250 Hz, 500 Hz, 1 kHz, 2 kHz, 3 kHz, 4 kHz, 6 kHz, and 8 kHz using headphones. When air conduction thresholds between 250 Hz and 4 kHz exceed normality levels of 20 dB HL, the bone conduction threshold will be measured at 250 Hz, 500 Hz, 1 kHz, 2 kHz, 3 kHz, and 4 kHz in order to distinguish between conductive and sensorineural hearing loss.

- Tinnitus analysis

Participants are asked whether they perceive the tinnitus unilaterally, bilaterally, or centrally, and whether the tinnitus sound is a pure tone, a noise, or a mixture of different sounds (polyphonic). Psychoacoustic characteristics of the tinnitus sound are assessed in a soundproof booth. As a psychoacoustic equivalent of frequency, the tinnitus pitch is obtained by use of a pitch matching technique. A two-alternative forced choice procedure will be employed using the contralateral ear as the reference ear. In cases where tinnitus is perceived bilaterally, the choice of ear is made arbitrarily. Using this technique, an attempt is made to identify the center pitch of the tinnitus. When multiple tinnitus sounds are perceived, patients are asked to focus on the most troublesome tinnitus sound. Pairs of pure tones (or noises in case of noise-like tinnitus), differing by one or more octaves, are presented to the individual, who has to indicate which of the stimuli best resembles the tinnitus sound. This procedure is repeated and finer adjustments are made to obtain the closest possible match of the tinnitus pitch.

Loudness is the perceptual correlate of sound intensity. The tone or noise defined as the pitch match is presented to the ipsilateral ear (when appropriate) and a loudness match is made by use of an alternating procedure. Because of compressed dynamic range frequently present at the tinnitus frequency, final loudness measurements are made with 1-dB steps. The absolute level of tinnitus loudness is measured in dB hearing level (dB HL). In addition, a calculation is made to provide a measurement of relative loudness expressed in dB sensation level (dB SL), which is defined as the level of the loudness match minus the auditory threshold at the tinnitus frequency.

- Changes in SPIQ and SPIN understanding

Speech comprehension in quiet is investigated using the Dutch NVA lists, which were developed by the Dutch Society for Audiology (Nederlandse Vereniging voor Audiologie, NVA) [40]. Each of the four lists consists of 12 monosyllabic words (consonant-vowel-consonant), of which the first one is used for training. The percentage of correctly identified items determines the speech recognition score. The lists are presented through headphones.

The Leuven Intelligibility Sentences Test (LIST) is used to measure speech reception in noise [41]. The long-term average frequency spectrum of the speech signal matches the spectrum of the noise. The noise level is constant at 65 dB SPL, while the sound level of the speech signal is changed according to the individual’s response. Lists of 10 sentences are presented through headphones. If the individual correctly repeats the keywords of a sentence, the sound level of the next sentence is decreased by 2 dB SPL. In case of an incorrect response, the level is increased by 2 dB SPL. The levels of the last five sentences of the list and the imaginary 11th sentence are averaged to acquire the speech reception threshold (SRT).

- Repeatable Battery for the Assessment of Neuropsychological status, adjusted for hearing-impaired individuals (RBANS-H)

Cognitive functioning will be evaluated by use of the RBANS-H [42]. The RBANS assesses five cognitive domains, i.e. Immediate Memory, Visuospatial / constructional, Language, Attention, and Delayed Memory, and consists of 12 subtests.

The domain Immediate Memory consists of two subtests. In the subtest List Learning, the same list of 10 words is presented over four trials, whereas the subtest Story Memory consists of a 12-item short story that is presented twice. After each presentation, the individual must recall as much of the words or the story as possible. Two subtests belong to the domain Visuospatial / constructional: a Figure Copy test, in which the individual is asked to copy a geometric figure, and the subtest Line Orientation, during which the individual needs to match two lines according to their orientation. The domain Language includes the subtests Picture Naming and Semantic Fluency. In the first subtest, 10 line drawings are to be named by the individual. In the second subtest, the individual is given 1 min to generate as many examples as possible from a certain semantic category. The subtests Digit Span and Coding contribute to the domain Attention. In the first subtest, a string of digits is presented, after which the individual is asked to repeat the digits in the correct order. In the subtest Coding, the individual is asked to complete a page of symbols with the corresponding digits according to a key on top of the page. The last domain, Delayed Memory, enquires how many items the individual recalls from the subtests List Learning, Story Memory, and Figure Copy.

The RBANS-H is developed especially for the purpose of examining the cognitive function of individuals with hearing impairment. To reach this purpose, a number of adjustments to the original RBANS have been made. By means of an accompanying PowerPoint presentation, written explanations are given to support the oral instructions and ascertain that the participant understands the instruction. In addition, all relevant stimuli are not only presented orally but also visually. All adjustments were made in accordance to the RBANS guidelines [43]. The total score before and after therapy will be compared in order to reveal any changes in different aspects of cognition due to HD-tDCS.

- Cortical auditory evoked potentials (CAEP)

Exploratory research is performed towards CAEP as a potential objective measure of therapy effect. Brain potentials will be elicited using an oddball paradigm during which the patient is instructed to press a button every time a rare stimulus (2 kHz) is presented in between frequent stimuli (1 kHz) through shielded headphones (Audio Technica ATH M30x Refaeds). Rare and frequent stimuli will occur with a probability of 20% and 80%, respectively, and the rise and fall time of the presented stimuli are both 5 ms. Stimuli are delivered by use of the Software Presentation™ (Neurobehavioral Systems, Inc). During this task, EEG is recorded (Micromed™ SD LTM 64 Express) using the interface “Gilat Medical TM Event Related Potentials system.” In total, 31 silver/silver chloride (Ag/AgCl) electrodes are placed according to the 10–20 Standard International Electrode System referenced to a chin electrode, with the ground electrode placed on the right mastoid. Vertical electrooculogram (EOG) is recorded using one electrode located below the right eye. After recording the EEG is sampled at 1024 Hz with 22-bit A/D resolution and band passed in the range of 0.02–450 Hz (Micromed™ SD LTM 64 Express).

All data will be analyzed by one researcher using Gilat Medical™ analysis software (Karkur, Israel). Recordings will be segmented into time epochs of 2 s which are time-locked to the stimuli. Baseline correction for each trial will be performed using the average of the 200 ms before stimuli onset for each channel separately. For each patient, all trials will be averaged according to the condition (target and non-target). An Independent Component Analysis (ICA) algorithm will be used to correct for external artefacts, such as eye blinks.

The analysis software will be used to calculate the area below the curve and the center of gravity of peaks within specific time windows in the average trace for the target condition. The time window for the different components is determined according to visual inspection of the average trace over all electrodes. Furthermore, latency average of the correct response is calculated as the time from stimulus onset to the time of the button press. Finally, a group average for the target and non-target records of the baseline measurement and the follow-up measurement is performed in order to compare the averaged response at baseline with the averaged response at the follow-up at three months.

### Sample size and power

The primary outcome is the change in TFI from baseline to follow-up at three months. A TFI decrease of 13 points is considered a minimal clinically important difference. Assuming a standard deviation (SD) of 20 points (SD found in international literature [44] as well as in a previous study of our research group [45]) and a significance level of 0.05, an achieved sample size of 39 per group is required to detect an effect of 13 points with 80% power using a two-sample t-test. Taking into account a possible 20% drop-out, 50 patients will be recruited into each group. Hence, a total of 100 patients will be recruited for the study.

### Assignment of interventions

#### Allocation

Participants will be randomized into the treatment group or the placebo group in a 1:1 ratio. The randomized list will be generated by an independent researcher using QMinim Online Minimization®. The allocation sequence will depend on the date on which participants are referred to the trial. Stratified randomization according to the grade of tinnitus severity on the TFI and gender will be performed. The TFI categorizes patients from grade 1 (slight tinnitus distress) to grade 5 (severe tinnitus distress). As patients with grade 1 are generally not in need of intervention, they will be excluded from the study (inclusion criterion: TFI > 24). Therefore, a total of eight strata will be defined by TFI grade and gender. Patient enrollment and allocation will be performed by an independent researcher.

In order to avoid any test bias, the researcher performing the baseline and follow-up measurements is blinded to the allocation of the patient. The investigator will be unblinded by an independent researcher after the three-month time point (T_1_). The independent researcher will be able to access the randomization sequence via QMinim, an online portal. For technical reasons, it is not possible to blind the investigator administering the HD-tDCS. Therefore, for each patient, the investigator administering the HD-tDCS will differ from the researcher performing the baseline and follow-up measurements.

#### Blinding

Both the patient and the investigator performing baseline and three-month follow-up measurements will be blinded up until and including the three-month time point (T_1_). Patients will be unblinded by an independent clinician at a separate hospital visit following the three-month follow-up time point.

### Data collection, management, and analysis

#### Data collection and management

OpenClinica LLC is used to enter and store data in a clean, secure, and efficient manner. This software package is developed especially for electronic data management in clinical research. Only the principal investigators have access to this password-protected database. Validation checks such as range checks for data values are programmed so that the number of mistakes is minimized. The information collected in this study is kept strictly confidential. Individual information and results are coded, with only the researcher knowing which code was assigned to each participant. The data are kept for 20 years.

#### Statistical methods

Data will be analyzed using SPSS statistical software version 25 (SPSS Inc., Chicago, IL, USA). The primary outcome is the change in TFI from baseline to three months after the treatment. A linear regression model using delta TFI (pre minus post therapy) as an outcome and treatment as a predictor with correction for age, gender, hearing loss, HADS score, and TFI grade will be used as primary outcome analysis. All patients will be used in an intention-to-treat (ITT) analysis.

Per-protocol analyses will be performed for all secondary outcome measurements to evaluate the treatment effect for the compliers. The change in secondary outcomes (which are all continuous variables) from baseline to follow-up at three months will be analyzed with a linear regression model with treatment as predictor controlling for age, gender, hearing loss, HADS score, and TFI grade. The questionnaires that are administered repeatedly over time will be modelled by a linear mixed model using patient as a random intercept to correct for repeated measures coming from the same patient. The Holm-Bonferroni method will be used to correct for multiple testing.

### Study monitoring

An independent Data and Safety Monitoring Board (DSMB) at the University Hospital of Antwerp will monitor the clinical trial for adverse events, adherence to the study protocol, and potential early stopping. The DSMB will meet twice a year and will biannually receive reports from the research team on patient safety, protocol deviations, data completion, and enrollment.

## Discussion

To date, several trials investigating the effects of tDCS on tinnitus severity have been published. Anodal stimulation of rDLPFC has been shown to positively affect tinnitus severity, but evidence is not conclusive due to large heterogeneity in study protocols and outcomes [46, 47]. Several RCTs have reported no significant effects of anodal stimulation of LTA on tinnitus severity, but many of these studies are underpowered and electrode placement is too variable to draw definitive conclusions [48, 49]. Overall, the degree of variability of protocols in these trials is considerable, with duration of tDCS sessions, number of sessions, applied current strength, and electrode placement varying greatly.

Furthermore, few trials on tDCS in tinnitus have sufficiently accounted for confounding factors such as age, gender, anxiety, depression, and the degree of hearing loss. Experimental and control groups are generally similar in mean age but are often not matched for gender. This may significantly confound trial results, as important gender differences in self-reported tinnitus severity have been found [50]. No systematic efforts have been made to control for the degree of hearing loss, although it has been suggested that tinnitus patients with less severe hearing loss may respond better to HD-tDCS [24]. Lastly, depression and anxiety may have a substantial influence on tinnitus outcomes and should be adequately controlled for. By recording hearing levels and assessing mental state by means of the HADS, we aim to take into account as many confounding factors as possible.

Recent research suggests that the stimulation of two sites within one tDCS session may have a more profound influence on the underlying corticospinal excitability than conventional single site tDCS [51]. These effects on neuronal excitability might then lead to longer-lasting effects on motor and cognitive performance [52, 53]. For these effects to occur, it is not necessary to stimulate both sites simultaneously [54]. Sequential stimulation of two target sites might be preferable to simultaneous stimulation, as the course of the applied current can be controlled more accurately.

To our knowledge, this is the first high-quality powered RCT to systematically investigate the effects of sequential dual-site HD-tDCS in chronic subjective tinnitus patients. Confounding factors such as gender, degree of hearing loss, anxiety, and depression will be controlled for. Additionally, outcome measures are carefully selected so that investigator bias is minimized. The results of this trial are expected to contribute to a definitive recommendation and consensus on the use of HD-tDCS in tinnitus treatment.

## Additional files

Additional file 1: SPIRIT 2013 Checklist: Recommended Items to address in a clinical trial protocol and related documents. (PDF 167 kb)

## Data Availability

According to the SPIRIT guidelines, the authors declare that data that break the blind will not be presented before the release of mainline results. The breaking of the blind will occur at the end of the study. A clinical article will be written on the primary (and including secondary) outcomes of the study, according to the CONSORT guidelines, and results will be disseminated regardless of the magnitude or direction of effect. The present trial is not industry-initiated; therefore, there are no publication restrictions imposed by sponsors. Data collected within the study is disseminated to the public through publications and lectures. The raw collected data will be stored locally after closing and checking the HD-tDCS database but will not be made publicly available.

## Abbreviations

CAEP: Cortical auditory evoked potentials
CONSORT: Consolidated Standards of Reporting Trials
EEG: Electroencephalogram
EOG: Electrooculogram
HADS: Hospital Anxiety and Depression Scale
HD-tDCS: High-definition direct current stimulation
HQ: Hyperacusis Questionnaire
HUI23: Health Utilities Index 23
ICA: Independent Component Analysis
LIST: Leuven Intelligibility Sentences Test
LTA: Left temporal area
RBANS-H: Repeatable Battery for the Assessment of Neuropsychological Status, adjusted for hearing-impaired individuals
rDLPFC: Right dorsolateral prefrontal cortex
SPIN: Speech-in-noise
SPIQ: Speech-in-quiet
SPIRIT: Standard Protocol Items: Recommendations for Interventional Trials
SRT: Speech Reception Threshold
SSQ12: Speech, Spatial and Qualities of Hearing Scale-12
tDCS: Transcranial direct current stimulation
TFI: Tinnitus Functional Index
TQ: Tinnitus Questionnaire
VAS: Visual analogue scale
